# A Survey on Design and Control Methodologies of High- Torque-Density Joints for Compliant Lower-Limb Exoskeleton

**DOI:** 10.3390/s25134016

**Published:** 2025-06-27

**Authors:** Jingbo Xu, Silu Chen, Shupei Li, Yong Liu, Hongyu Wan, Zhuang Xu, Chi Zhang

**Affiliations:** 1 School of Medical Devices, Zhejiang Pharmaceutical University, Ningbo 315500, China; xujb@zjpc.net.cn; 2 Ningbo Institute of Materials Technology and Engineering, Chinese Academy of Sciences, Ningbo 315201, China; lishupei@nimte.ac.cn (S.L.); liuyong@nimte.ac.cn (Y.L.); wanhongyu@nimte.ac.cn (H.W.); 3 Department of Electrical and Electronic Engineering, University of Nottingham Ningbo China, Ningbo 315100, China; zhuang.xu@nottingham.edu.cn

**Keywords:** exoskeleton, series elastic actuator, flexible joint, harmonic drive, torque motor, permanent magnet synchronous motor, impedance control, human-robot interaction

## Abstract

The lower-limb assistance exoskeleton is increasingly being utilized in various fields due to its excellent performance in human body assistance. As a crucial component of robots, the joint is expected to be designed with a high-output torque to support hip and knee movement, and lightweight to enhance user experience. Contrasted with the elastic actuation with harmonic drive and other flexible transmission, the non-elastic quasi-direct actuation is more promising to be applied in exoskeleton due to its advanced dynamic performance and lightweight feature. Moreover, robot joints are commonly driven electrically, especially by a permanent magnet synchronous motor which is rapidly developed because of its compact structure and powerful output. Based on different topological structures, numerous research focus on torque density, ripple torque suppression, efficiency improvement, and thermal management to improve motor performance. Furthermore, the elaborated joint with powerful motors should be controlled compliantly to improve flexibility and interaction, and therefore, popular complaint control algorithms like impedance and admittance controls are discussed in this paper. Through the review and analysis of the integrated design from mechanism structure to control algorithm, it is expected to indicate developmental prospects of lower-limb assistance exoskeleton joints with optimized performance.

## 1. Introduction

The lower-limb exoskeleton has emerged as a transformative technology with profound implications for enhancing human capabilities in various domains: military, industrial, medical, and personal applications [1,2,3]. The lower-limb exoskeleton can be functioned for sport enhancement, sport endurance, sport assistance, and sport rehabilitation [4,5]. As it is a crucial component, the drive joint assembles different linkages and achieves motion in multiple degrees of freedom. Meanwhile, it takes up most of the exoskeleton’s weight and determines the dynamic performance. In this survey, the actuation joint is intended to aid the hip and knee movements of elderly individuals and those with motor dysfunction, and therefore, the drive joint is designed to have excellent power density for movement assistance and outstanding stability for user experience and safety [6].

Such assisted joints are mainly driven by a pneumatic system or electric motor [7]. Compared to the pneumatic actuator which requires an attached air compressor, the electric motors are directly embedded inside joints, consequently reducing difficulties in portability and wearability [8]. The motors commonly utilized in lower-limb exoskeleton joints comprise a brushless direct current (BLDC) motor, alternative current servo motor (ACSM), and permanent magnetic synchronous motor (PMSM). Specifically, the PMSM has recently gained significant attention and adoption due to its inherent advantages in its compact and lightweight structure [9]. Aiming to support the exoskeleton joint to accomplish squatting and walking movements, the PMSM is expected to have high torque density and efficiency. Moreover, the corresponding torque ripple and operational thermal problem should be considered to improve the stability and safety of joints, thereby achieving the functionality and interactivity of exoskeletons for hip motion assistance.

Furthermore, the elaborated joint with a high-performance torque motor requires compliant control algorithm to create safe and stable interaction between exoskeleton joint and human body [10]. Compared to conventional control methods, the complaint control system has better ability to mimic human-like motion with flexibility and adaptability. Through the establishment of sophisticated model and consideration of physical properties and constraints, the joint can adjust its stiffness and displacements in response to external forces and the user’s natural motions, thereby enhancing comfort and safety. Extensive research and review have been conducted on design for actuation joints within lower-limb-assisted exoskeletons [11,12,13], but fewer articles concentrate on the integrity survey of a skeleton joint from mechanism design to control methods. Aiming to propose a comprehensive survey on recent research of joints for lower-limb-assisted exoskeletons, this survey firstly presents an analysis of the demand and design requirements specific to exoskeleton actuation joints, followed by a thorough review and comparison of different elastic and non-elastic designs. It also discusses innovative research and methodology concentrating on performance improvement for PMSMs utilized in exoskeletons with various topological structures. Eventually, it analyzes distinct compliant control methods and introduces their corresponding latest development.

## 2. Design Requirements for Joints of Low-Limb Exoskeleton

Based on human biomechanics, the configuration of an exoskeleton is compared to that of the human lower limbs. The study focuses on the motion plane, motion joint, walking mechanism, and driving joint to determine the motion range and output torque of each driving joint. In the study of human movement, the human body is divided into three separate planes: sagittal, coronal, and transverse. The movement of human joints in the direction of three planes are defined as flexion/extension, abduction/adduction, and internal/external rotation [14]. The joints of the human lower limb are mainly the hip, knee, and ankle joints. Among them, the hip and ankle joints have a certain range of motion in all three planes, while the knee joint has only sagittal flexion and extension. Human gait is repetitive and cyclical, and during the gait process, the human lower limbs can be divided into two basic states: the stance phase and the swing phase [15]. Table 1 shows the movement pattern, range of motion, and peak torque of each joint when a human is in a normal walking state. Subsequently, the degrees of freedom of the exoskeleton’s actuator joints can be determined, and the magnitude of the output torque provided by the actuator joints can be selected without restricting the wearer’s range of motion. As shown in Table 1, the hip joint requires a greater drive torque than the knee joint when walking on level ground. A greater drive torque generally corresponds to a greater joint mass. Therefore, when designing hip and knee joints, the mass of joint and the torque requirement should be balanced.

The requirements of the actuation joints of the gait-assisted exoskeleton robot are clarified. To meet the functional requirements of walking-assisted exoskeleton robots, it is essential to couple the exoskeleton robot to the wearer’s lower limbs for movement and consider the exoskeleton‘s performance in terms of comfort, safety, and weight. Thus, the design requirements for actuated joints in exoskeletons are as follows:

Wide range of torque output. This capability allows exoskeleton joints to adapt to diverse environments (e.g., uphill versus flat-ground walking) by adjusting torque in real time to counter gravity and movement resistance [16]. It also provides personalized assistance for users with varying physical abilities, from mild muscle fatigue to severe movement impairments, ensuring tailored support for daily activities. Furthermore, it stabilizes natural body coordination during actions like standing or stepping, enabling smooth synchronization between robotic assistance and human motion without compromising flexibility or causing abrupt force conflicts [17].

Lightweight. Lightweight drive joints enable robots to operate more efficiently during movement. This is particularly beneficial for battery-powered exoskeleton systems, as a lightweight joint design can significantly reduce power requirements, thereby extending the battery life and improving the robot’s endurance. Furthermore, lightweight joints can respond more quickly to the wearer’s movements, providing a natural and smooth movement experience. As a result, a lighter joint design improves the motion flexibility of exoskeleton robots while reducing the burden on the user and minimizing fatigue. In addition, from a mechanical design perspective, lightweight joints typically require advanced materials and designs to ensure sufficient structural strength and durability for stable operation of exoskeleton robots in various scenarios.

High bandwidth. Joints with high bandwidth provide fast, precise motion control and response. It allows the joint to quickly sense and respond to commands from sensors or control systems. More precise control which accurately mimics human movement and provides needed assistance can be achieved with high bandwidth actuators. It improves the dexterity, safety, and stability of the exoskeleton. In addition, the high bandwidth allows the exoskeleton to adapt to the changes in the wearer’s needs, such as rapidly adjusting the output torque angle for different terrain.

Back-drive capability. The exoskeleton’s joint can respond to the user’s movements and adjust motion path through back-drive capability. Such feature helps the robot better adapt to the wearer’s movements, reducing unnecessary resistance and discomfort, and ultimately improving the wearer’s comfort. Especially in emergency situations when the user wants to instantly adjust their gait, the back-drive ability allows the joint to be more flexible. Additionally, the back-drive capability reduces the resistance of the exoskeleton robot to the drive system during the user’s movements, thereby reducing energy consumption. It is crucial to prolong battery life and enhance the efficiency of walking assistance.

Robustness and dexterity. These two requirements ensure exoskeleton joints withstand unpredictable physical interactions (e.g., uneven terrain or accidental collisions) while maintaining precise motion control for tasks like stair climbing or obstacle avoidance. Robustness guarantees structural integrity under prolonged operational cycles and environmental extremes (e.g., moisture, dust), whereas dexterity enables adaptive limb coordination to match natural human kinematics, preventing rigid constraints that restrict free movement or induce user fatigue. Together, they balance system durability with responsive biomechanical compatibility, critical for safe and intuitive human–robot collaboration across daily living and rehabilitation scenarios.

## 3. Mechanism Design of Lower-Limb Exoskeleton Joint

Based on the design requirements discussed in the last section, the mechanism design of exoskeleton joint is conducted to achieve specific functional, performance, and reliability, which involves selecting appropriate mechanical structures, transmission units, and actuators to meet the necessary design requirements.

In the field of mobility-assisted exoskeletons, actuation methods are currently mainly classified into motorized actuation, hydraulic actuation, and artificial pneumatic muscle actuation [15]. Motorized actuation technology is more mature and widely used in exoskeleton compared to hydraulic and pneumatic actuators [18]. A motorized actuator has the advantages of simple control, fast response, and easy to ensure the control accuracy, and at the same time, its maintenance and use, signal monitoring, transmission, and processing are very convenient. The motor actuator joints are mainly divided into three types: joints with harmonic drives, other (series or parallel) elastic joints, and non-elastic joints with quasi-direct drives.

### 3.1. Joints with Harmonic Drives

Conventional actuators often rely on high-speed, low-torque motors, and high-reduction harmonic drives with a ratio larger than 50:1. This approach is commonly used in industrial robots and early exoskeletons, like the HAL series developed by the University of Tsukuba. The HAL exoskeletons, made of aluminum alloy and rigid materials, adopt DC motor harmonic drives to power knee and hip joints and passive spring joints for ankle joints to maintain the relative stability of the human body [19]. Subsequent iterations employ flat-type brushless DC motors coupled with harmonic reducers for single-leg joint actuation. Angular measurements are typically obtained through high-resolution optical encoders and Hall-effect sensors [20,21]. The GEMS hip-assisted exoskeleton robot, created by the Samsung Advanced Institute of Technology, uses a compact brushless DC motor actuator equipped with multi-stage gears. It is fastened around the waist with hip and thigh straps to aid hip joint movement [22]. A lower-limb exoskeleton developed by the University of Science and Technology of China (USTC) incorporates brushless DC motors paired with harmonic accelerators to facilitate gait assistance in rehabilitation training scenarios [23]. The UEXO series exoskeleton developed by the University of Electronic Science and Technology (UEST) features joints that utilize disc motors along with harmonic reducers, enabling efficient achievement of specified control objectives [24]. Additionally, a knee exoskeleton employs a four-bar linkage mechanism to simulate the patellar motion of human knee joints, driven by an electric motor coupled with a harmonic reducer [25]. Table 2 provides a comparative analysis of key technical specifications across representative harmonic drive-based exoskeleton systems, including actuator configurations, reduction ratios, torque capabilities, and mass distribution characteristics.

Although the harmonic reducer can achieve high-output torque in a compact space, it has low back-drive efficiency and high mechanical impedance. The motor end cannot sense the movement changes in the human leg, which affects the ability of the exoskeleton to interact with the human body in a bidirectional manner. In addition, the harmonic reducer has poor adaptability. The substantial mass and volume of the harmonic drive joints conflict with the critical need for lightweight exoskeleton designs.

### 3.2. Other Series, Parallel or Hybrid Elastic Joints

**Figure 1:**
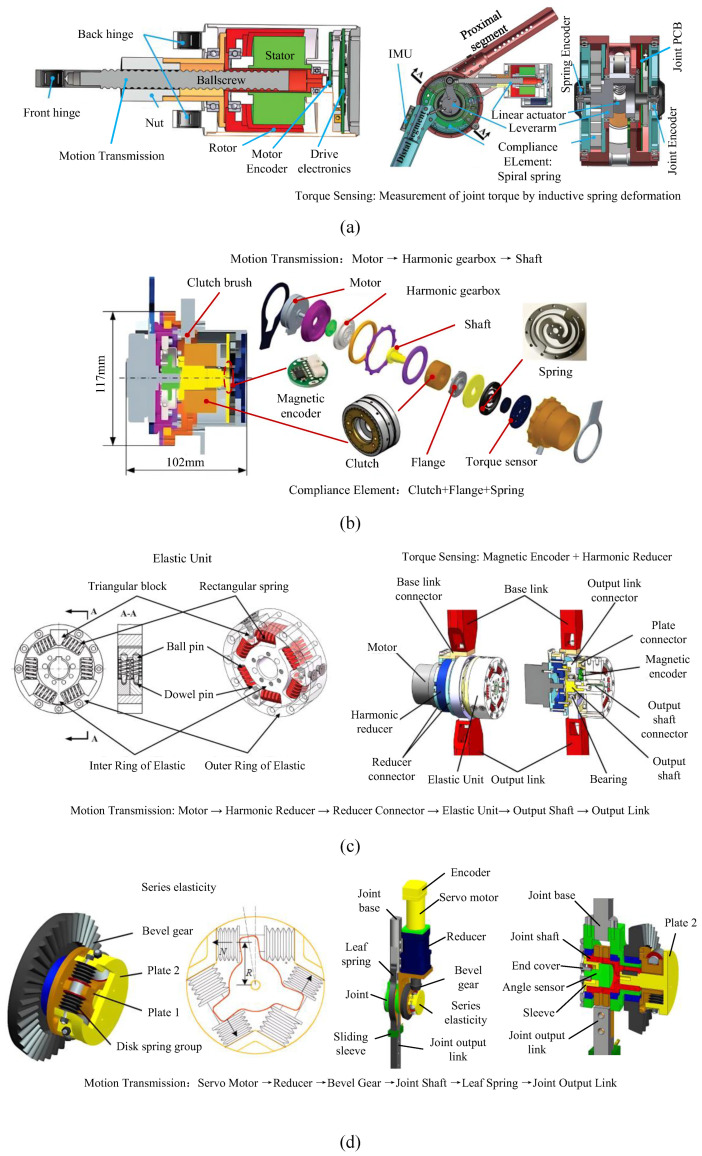
Various designs of elastic joints. (**a**) The linear actuator integrating a brushless DC motor, a ball screw, and an encoder; (**b**) the clutched series elastic actuated hip joint; (**c**) the novel parallel SEA including brushless DC motor, harmonic reducer, elastic unit, magnetic encoder, base connection, and output link; (**d**) the hip flexible joint comprising a series elastic drive module and a parallel elastic module.

In recent years, various designs of joints with fixed or variable stiffness which often refer to as series elastic actuators, have been proposed. These joints are based on conventional actuators by using elastic components connected between the motor output and the load. The elastic components increase the flexibility of the actuator joints and reduce the impedance, which improves the adaptability of the robot joints and is widely used in fields such as prosthetics [26]. In the field of exoskeletons, tandem elastic actuation is commonly applied to individual hip or knee joints, with several combinations available. It is important to maintain objectivity and avoid biased viewpoints when discussing these designs. Two typical designs are linear series elastic actuation (SEA) [27,28,29] and rotary series elastic actuation (RSEA) [30,31,32,33]. A single-degree-of-freedom exoskeleton, named RoboKnee, employs ball screws and compression springs to assemble a servo-motor drive assembly and a rigid output assembly into a linear tandem elastic actuator that generates torque around the knee. In another example, the Mindwalker exoskeleton joint uses a specific linear actuator and torsional tandem spring to form a tandem elastic actuator. Figure 1a shows the linear actuator which integrates a brushless DC motor, a ball screw, and an encoder. Its unique hollow structure is designed to allow the ball screw to pass through the center of the motor stator, allowing the linear actuator to be more compact A high-power serial elastic joint comprises a frameless brushless motor, harmonic reducer, and two-spoke torque spring, and it integrates both a torque sensor and two encoders to measure motor angle and torque deformation, which is suitable for a torque-controlled exoskeleton [34]. A clutched series elastic actuated hip joint is presented, as depicted in Figure 1b, which incorporates a planar brushless motor, a harmonic gearbox, an electromagnetic toothed clutch, and a disc-shaped torsion spring [35].

While all the designs employed elastic units in series with brakes, recent advancements have led to the development of parallel elastic actuators (PEAs) that cater to specific task requirements. Furthermore, there have been efforts to combine both series and parallel elastic units in certain actuators. A novel parallel elastic actuator is designed with a separated-component structure and double parallel springs [36]. The structure includes brushless DC motor, harmonic reducer, elastic unit, magnetic encoder, base connection, and output link, as shown in Figure 1c. A novel coupled variable parallel elastic actuator is proposed to actuate exoskeleton hip joints in the sagittal plane, consisting of a brushless motor and planar coil springs in parallel [37]. A modular knee actuator combines the advantages of both series and parallel elastic units [38]. A flexible joint is designed to fit the hip joint, comprising a series elastic drive module and a parallel elastic module [39], as shown in Figure 1d. The series drive module includes a brushless DC motor, an incremental encoder, a planetary gear reducer, an angle sensor, and a butterfly spring, while the parallel elastic unit uses a leaf spring. To systematically compare these elastic joint designs, Table 3 summarizes their key characteristics, including actuator type, core components, target joints, and elastic configurations.

In general, the tandem elastic actuators enhance joint adaptability and safety through elastic components that reduce impedance, absorb shocks, and enable compliant motion while simultaneously improving energy efficiency via dynamic storage and release of elastic energy [40,41]. However, the drive system’s complex structure, large joint mass, and poor responsiveness of elastic components to high-frequency motions result in low bandwidth and poor dynamic performance. It limits the ability to cope with the complex and rapid changes in practical working conditions.

To mitigate the negative effects of elastic elements on high-frequency joint motions in series elastic actuators (SEAs), recent studies have proposed several strategies. These include optimizing the stiffness and structure of elastic components, such as using nonlinear or variable stiffness springs [42,43,44,45,46], and introducing parallel elastic elements or damping [47,48,49] to distribute high-frequency loads and reduce high-frequency jitter. Additionally, advanced control strategies, such as feedforward compensation, state observers, and adaptive impedance/admittance control, can dynamically adjust system parameters in response to variations in gait frequency. These techniques collectively enhance the motion bandwidth and tracking accuracy of lower-limb exoskeleton joints under dynamic conditions.

### 3.3. Quasi-Direct Actuators

**Figure 2:**
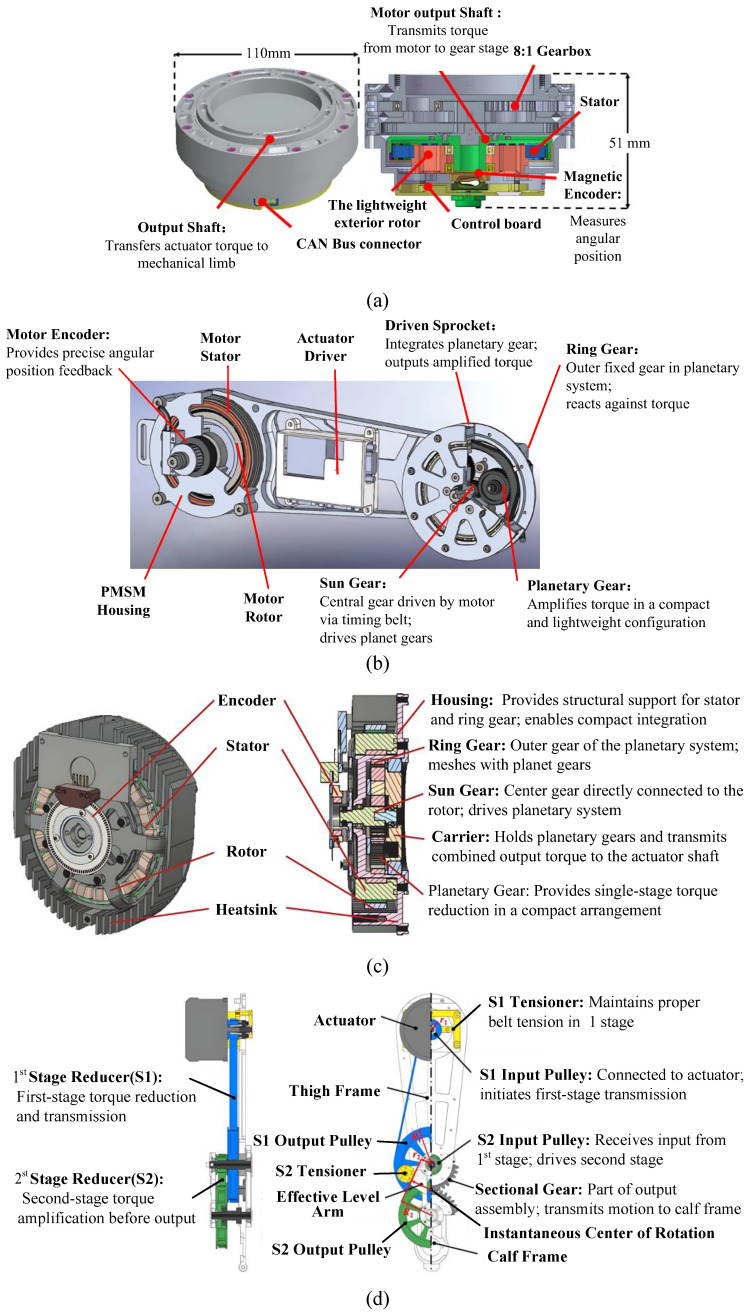
Various designs of the quasi-direct drive joints: (**a**) the joint with high-torque motor and an embedded planetary gear reducer; (**b**) the knee and ankle power exoskeleton; (**c**) the joint with custom brushless DC motor with package windings; (**d**) the joint with the installed motor at the near end of the thigh frame.

To enhance suppleness, tandem elastic actuators must make trade-offs in bandwidth, making the control problem more challengeable. This limitation is especially significant for multi-jointed lower-limb exoskeletons, which involve repetitive, highly interactive forces with the body during movement and are subject to disturbances from changing gaits and varying terrain conditions. Most quasi-direct actuators use motors with high torque density coupled to planetary gears with low reduction ratios. This technology was initially applied in the field of footed robots [50] and prosthetics. However, they do not meet the requirements of lower-limb exoskeleton joints in terms of mass, torque, and speed. Recently, inspired by such technology, some quasi-direct drive joints for exoskeleton robots have also been studied. A quasi-direct drive joint, seen in Figure 2a, integrates a high-torque motor and an embedded planetary gear reducer [51,52]. A drive module integrates a frameless motor with the mechanical structure of the knee and ankle exoskeleton [53], as illustrated in Figure 2b. A synchronous belt connects the motor output shaft to the sun gear, and the planetary gear is built into the driven chain, resulting in a lightweight and power-intensive actuator. The impact of the package on torque density is investigated [54], and a custom brushless DC motor with package windings is developed, as shown in Figure 2c. They used a sun-planet gear reducer with a smaller gear ratio (7:1) and shared support components. A rolling knee joint structure utilizes a two-stage planetary gear reducer and a synchronous belt drive to amplify the torque of the customized motor and meet the auxiliary requirements [55]. As shown in Figure 2d, it allows the motor to be installed at the near end of the thigh frame, significantly reducing the exoskeleton’s inertia. In summary, the quasi-direct drive has the advantages of being lightweight and having good dynamic performance while producing high torque. It also allows for energy regeneration to improve motion efficiency. However, its motion accuracy is affected by the difference in gear meshing return, and there is significant heat loss [56]. To enhance suppleness, tandem elastic actuators face trade-offs in bandwidth, complicating control challenges, particularly in multi-jointed exoskeletons subjected to dynamic interaction forces and environmental disturbances. As the gear ratio is intended to be low in the quasi-direct drive joints, how to further increase the torque density of motor becomes crucial for lightweight design and faster active-drive response under such semi-direct drive applications. Table 4 summarizes key quasi-direct drive designs, highlighting their configurations, target joints, and performance trade-offs.

To enable a clear comparison of actuator types, Table 5 summarizes representative joint designs and highlights their differences in torque density, back-drivability, dynamic bandwidth, and mechanical complexity. Based on the comparative evaluation presented in Section 3.1–Section 3.3, quasi-direct drive actuators emerge as the most promising option for lower-limb exoskeletons, particularly given their stringent requirements for compactness, lightweight structure, high torque output, and dynamic responsiveness. Quasi-direct drive actuators provide high torque density and fast dynamic response due to low gear ratios and high-performance motors, enhancing control bandwidth and real-time responsiveness. Their inherent back-drivability ensures safer and more natural human–robot interaction, while their structurally simple design reduces overall weight and facilitates modular integration. In contrast, harmonic drives exhibit high mechanical impedance and poor back-drivability, and series elastic actuators introduce compliance-induced latency and increased complexity. Moreover, quasi-direct drive actuators support effective thermal management and precise control optimization, making them highly suitable for dynamic locomotion. These advantages collectively suggest that quasi-direct drive joints offer an optimal trade-off between performance, reliability, and integration and have become a key focus in the development of next-generation exoskeleton systems.

To further enhance quasi-direct drive joint performance, two major optimization directions are identified: motor advancement and gear mechanism refinement. On the motor side, high-torque-density permanent magnet synchronous motors (PMSMs), particularly those using axial flux and dual-stator radial flux topologies, are adopted for compact and powerful actuation. Techniques such as fractional-slot windings and multilayer magnetic circuits improve the torque-to-weight ratio. On the gear side, low-ratio planetary reducers (5:1 to 20:1) ensure low mechanical impedance and high back-drivability, while compliant planetary carriers eliminate backlash and improve transmission accuracy. Additionally, thermal losses are mitigated through segmented rotor designs, phase-change materials, and lumped thermal modeling, enabling stable operation under high-load conditions. These are discussed in the next section. Notably, a new bidirectional drive joint architecture incorporating a compliant planetary carrier into its back-drive mechanism [57] achieves simultaneous backlash elimination and load equalization across planetary gears. This design represents a promising solution to key technical challenges in the development of lower-limb exoskeleton joints.

## 4. Motor Design of Lower-Limb Exoskeleton Joints

Aiming to drive the actuators to accomplish the movement of exoskeleton actuation joint efficiently and safely, a permanent magnetic synchronous motor (PMSM) is commonly designed and adopted due to its inherent advantages in compact and lightweight structure. Such motors are further improved toward better electromagnetic performance based on their topological structure, including the magnetic flux direction and the number of stators and rotors. This section firstly investigates various PMSMs with different topological structures and subsequently provides profound knowledge for motor performance optimization.

The exoskeleton joint commonly operates at low speed with high torque level, and the form factor of humanoid joint is restricted, so it is necessary to promote the output torque density of drive motor. At the same time, the torque ripple is expected to be eliminated as it causes vibration and impacts the targeting precision and user comfort. Moreover, the energy losses mainly resulted by eddy current and iron wear are potential to restrain machine efficiency. Efficiency is also affected by the heat generated during the motor operation which intensifies iron and copper losses. Additionally, the rising temperature causes stability and safety problems in motors. Aiming to satisfy the design requirement of exoskeleton discussed in Section 2, the utilized PMSM should be optimized to improve its capacity in the torque density, stability, efficiency, and safety.

### 4.1. Topology

According to the distinct magnetic flux direction, PMSM utilized in exoskeleton can be classified into axial flux permanent magnet machine (AFPMM, also called disk-type motor) and radial flux permanent magnet machine (RFPMM). Specifically, AFPMM is increasingly applied in exoskeletons, because its pancake shape and high power density provides the joint with smaller volume and higher efficiency [10]. Nevertheless, the recent innovation of RFPMM has generally overcome the drawbacks of conventional radial flux machines and verified the improved capability of application in robotic joints.

**Figure 3:**
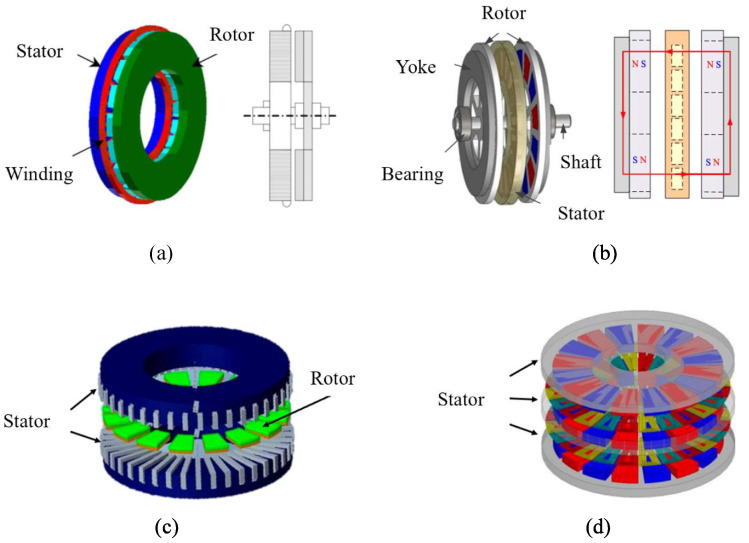
Single-sided structure (**a**); double-sided rotor internal stator structure (**b**); double-sided stator internal rotor structure (**c**); multi-disk structure (**d**).

Axial flux permanent magnet machine. Based on the relative position and number of rotor and stator assembled in the machine, AFPMM has four types of structure: single-sided structure (SS), double-sided rotor with internal stator structure (also named as “TORUS”), double-sided stator with axial flux internal rotor (AFIR) structure, and multi-disk structure (MS) [58]. As displayed in Figure 3a, the SS machine has the simplest topological structure, where thrust bearings are commonly necessary to prevent axial displacement in the rotor since the stator acts as the magnetic circuit for rotating magnetic poles. Although such simple structure makes it easier to manufacture and maintain, the alternating magnetic field generated by the rotor within the stator can result in losses, which are potentially the main sources of performance limitation [59]. It is concluded that the SS machine had nearly identical performance with dual-rotor machine under low electric loadings but dropped more dramatically with increasing loadings due to its poor heat dissipation [60]. Thus, numerous engineers are attracted to design multi-stage machines which have higher performance and space utilization. The TORUS machine has a sandwich shape where the single stator exists between dual rotors, and therefore, it can optimize the utilization of windings and air-gap magnetism, allowing for efficient operation. Moreover, it effectively mitigates the axial magnetic pull force, thereby simplifying the mechanical design of the motor and enhancing convenience in implementation. As shown in Figure 3b, a TORUS motor with ironless stator was designed [61]. It adopted the single-layer concentrated windings to decline the air-gap length, while the plate-shaped permanent magnet strengthens the air-gap magnetic field. The AFIR machine has a similar structure but with the inner rotor. Different from TORUS machine, the main flux does not travel through the single rotor so that a steel disk is not adopted. The double stators generate double windings, which enhances the power density, allowing for a higher power output in a compact design. The dual-stator PMSM shown in Figure 3c can be regarded as the combination of the permanent magnet arc motor and the permanent magnet stator motor [62]. It demonstrated that the unit with 6 slots and 11 rotor poles performs higher output torque and lower torque ripple. Aiming to further improve the performance of AFPMM in limited space, the multi-stage machine with N stators and (N + 1) rotors has been developed. The addition of rotors and stators can increase the amplitude of air-gap magnetic density and consequently increase the electromagnetic torque with the unchanged permanent magnet mass and machine size. Figure 3d illustrates the AFPMM with three rotors and two stators [63]. The machine optimally utilized the winding and air-gap magnetic while effectively managing axial magnetic tension. In conclusion, the addition of stators and rotors generates more magnetic flux paths and electromagnetic interaction interfaces, thereby improving motor output torque and operation efficiency. However, the complicated structure also results in greater manufacturing challenge, larger weight and more operational problems due to cross-coupling and leakage of magnetic flux. It is significant to find the optimal number of stators and rotors for the specific joint motor.

**Figure 4:**
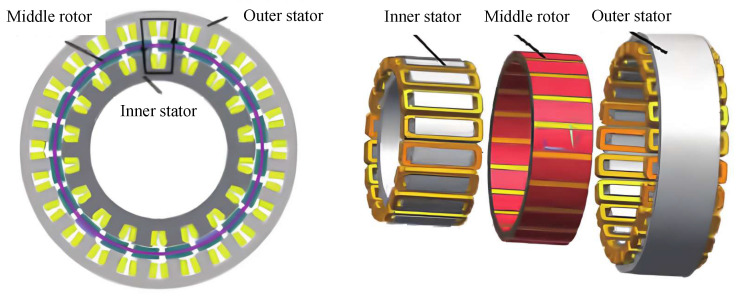
The RFPMM with single rotor and double stators.

Radial flux permanent magnet machine (RFPMM) has been widely utilized in robotics for decades, and numerous designs have been proposed for exoskeleton joints which require higher electromagnetic output within smaller form factors. Similar with AFPMM, RFPMM can be commonly classified into single-stator structure and multi-stator structure. The conventional single-stator RFPMM has lower manufacturing and maintenance costs because of the complete industrial chain and mature manufacturing process. Through radial magnetic flux direction design, the short magnetic circuit and high magnetic flux utilization rate provide the motor with relatively high efficiency and electromagnetic output. For instance, a well-designed single-stator RFPMSM designed for robotic joint has an average torque output of 14.94 Nm and maximum ripple of 0.23 Nm with optimal winding configuration [64]. However, the limitation number of flux path restricts further improvement in motor performance. Thus, more stators are added to increase number of air gap and flux path to improve torque density. Through such design, the motor internal space is better utilized to minimize cogging torque and strengthen dynamic response capacity. Furthermore, the two stators are driven by two independent controllers, which can flexibly allocate power of stators according to operating conditions and adopt different control strategies to achieve high-precision control. As shown in Figure 4, a dual-stator RFPMM was designed for high-torque-density joint [65]. The addition of stator increased magnetic flux density, and the optimization of both inner and outer PM size also enhanced the electromagnetic performance. The experimental results stated that contrasted with the single-stator motor of the same size, the unit torque of dual-stator motor was increased. It is concluded that the conventional single-stator RFPMM has moderate and stable performance with low manufacture and maintenance cost, while the dual-stator RFPMM has better electromagnetic performance but increases difficulties of design and control strategies.

### 4.2. Performance Optimization

Based on the superior designed topological structure, the capability of PMSM can be potentially promoted to improve robotic feasibility and user experience. In total, four types of performance metrics are discussed in this paper. Primarily, the torque density is the fundamental optimization metric because it is significant to increase output electromagnetic torque in limited inner space to support flexible and stable motion of exoskeleton joint. Secondly, the corresponding ripple torque should be reduced because it results in mechanical vibration which quickens the wear and tear of machine. Such vibrations also decorate control stability and user comfortability under a high load. Moreover, efficiency is another metric that should be considered. Thus, it is important to reduce motor loss mainly including copper loss, iron loss, and eddy current loss. Finally, temperature rise should be considered by identifying the thermal characteristics of PMSM to restrain the operational temperature rising and further improve the reliability.

To establish efficient and accurate mathematical model between multiple optimization objectives and numerous topological parameters, the current research tends to adopt the accurate subdomain method. It divides the entire field into multiple subdomains based on material properties, excitation types, and field shapes [66]. The partial differential equations satisfied by each subdomain are derived from the Maxwell equation system. Moreover, multiple parameters raise the calculation time and difficulty of PMSM optimization. The sensitivity test can not only quantitatively obtain the influence of parameters on optimization targets but also divide parameters into different groups according to the degree of impact to achieve dimension reduction [67]. Additionally, the optimization objectives tend to conflict with each other, so it is significant to adopt the Pareto solution. Unlike differential evolution, particle swarm optimization, and genetic algorithms, Nondominated Sorting Genetic Algorithm II (NSGA-II) effectively avoids the slow convergence and premature convergence associated with local optima and further enhances the robustness of multi-objective optimization models [65].

Torque density improvement. In exoskeletons, the joints typically operate at low speeds, yet they demand high torque levels. Moreover, multiple joints cooperate to accomplish robotic walking and squatting, and the motor is expected to undergo miniaturization while increasing the electromagnetic torque. The common methodology mainly focuses on improving motor space utilization and changing winding methods. An outer-rotor PMSM adopts the fractional slot concentrated winding which minimizes the end winding height and results in a more compact structure [68]. It also has a relatively large slot number (36 slots) to maintain balance between torque output and stamping difficulty. Combined with a planetary reducer featuring a 10:1 speed reduction ratio, the output torque density is apparently increased. Moreover, the torque density of a PMSM with the same number of slots and poles is promoted through the optimization of PM geometry and split ratio [69]. Additionally, a dual-stator motor is designed to improve motor inner space utilization [70]. Based on the air gap magnetic flux density calculation of both inner and outer stators, their parameters are adjusted correspondingly to mitigate the complex problem that arose from coupling magnetic fields and armature reactions. Consequently, the output torque density of such dual-stator machines is increased. Overall, winding method improvement and stator addition collectively improve motor torque density, thereby enhancing its suitability for compact and high-torque exoskeleton robot joints.

Torque ripple suppression. The presence of harmonics in the motor’s magnetic field is a significant contributor to torque ripple. Specifically, the cogging torque generated by magnetic interaction between stator and rotor is the main source of torque ripple. Such torque induces fluctuations in the robotic joint motion, consequently declining accuracy and degrading user experience. It can be eliminated from two aspects, optimization of topological structure and iteration of control algorithm. A double-sided axial flux machine combined with current harmonic injection has an air-core configuration with concentrated windings [71]. Such design eliminates the stator and rotor magnetomotive force harmonics, thereby suppressing the torque ripple. An analytic model and feedforward control algorithm are established to predict the magnetomotive force and reduce torque ripple. To conquer the limited accuracy of harmonic current injection method, a torque ripple surrogate model being independent on motor parameters is proposed to quantify the association between torque ripples and stator currents [72]. The experimental outcomes verified that the torque ripple was effectively decreased while the machine parameters are not known, so that this method had broad prospects for application. In summary, structural design innovations and advanced control strategies have demonstrated strong potential in effective mitigating torque ripple, thereby improving motion smoothness in exoskeletal applications.

Efficiency improvement. The permanent magnet creates both axial and circumferential magnetic flux in the air gap. As the rotor disc carries the permanent magnet, the alternating magnetic field induces eddy currents in adjacent conductors. At higher frequencies, these induced currents lead to increased motor losses and reduced efficiency [73,74]. Moreover, the iron loss deeply related to the magnet material is also an obstacle to improving motor efficiency. Therefore, it is essential to implement measures that monitor and minimize eddy current losses and iron losses to improve machine efficiency. Some studies explore the change rule of eddy current loss [75,76,77]. It is summarized that the eddy current loss is lower as magnet thickness in shaft direction decreases and the frequency of pulse width modulation (PWM) ripple current decreases. Moreover, the choice of segment rotor structure is proved to have an influence on the suppression of rotor eddy current loss [78]. When the rotor surface is interrupted by non-conductive segments or slots, these segments break the continuity of the conducting material, reducing the path for circumferential eddy currents to flow. As a result, eddy current losses are decreased. The experimental results show that rotors with carbon fiber in sleeve axial segmentation could reduce eddy current losses [79], as indicated by iron loss curve of RFPMM with an amorphous metal stator core. In summary, optimizing magnet design and rotor segmentation are key strategies to mitigate eddy current and iron losses and improve motor efficiency, thereby enhancing the stability and battery life of lower-limb exoskeleton joints.

Thermal characteristics optimization. During the motion of exoskeleton joint, the electric loss caused by the winding resistance and mechanical friction between operational connection components will increase the machine temperature. Such a problem not only leads to energy loss in the form of heat but also impacts the long-term durability of the motor components. To achieve stable and safe control of drive motors, it is important to establish a heat management system to figure out the thermal characteristics of machines and further mitigate the problem of energy loss and temperature rising. A lumped parameter thermal network (LPTN) decomposes complex thermal systems into discrete lumped nodes, where each node represents a region with uniform temperature. These nodes are interconnected through thermal resistances, thermal capacitances, and heat sources, forming an equivalent thermal network [80]. A LPTN of PMSM with a robot link analyzes the sources of heat including core loss, copper loss, and mechanical loss, which lays foundation for improving efficiency and reducing motor temperature [81]. Another LPTN established for high-torque-density PMSM effectively analyzes the heat sources and heat transfer performance so that temperature distribution in the motor is predicted [11,35]. Furthermore, phase-change material (PCM) exhibits significant advantages in motor thermal management by utilizing its latent heat absorption properties during the phase change process [82]. PCM is filled into the motor case to suppress transient temperature rise, and the numerical simulation results demonstrate that a small amount of PCM effectively declines the temperature rising under frequent high overload conditions [83]. In conclusion, the integration of LPTN modeling and PCM-based thermal management systematically monitors heat distribution in motor and further suppresses the temperature rise, consequently ensuring efficiency and durability in lower-limb exoskeleton joints with high power density.

## 5. Compliant Control of the Lower-Limb Exoskeleton Joint

**Figure 5:**
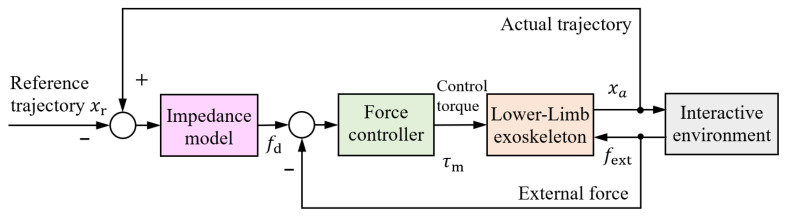
Impedance control structure.

**Figure 6:**
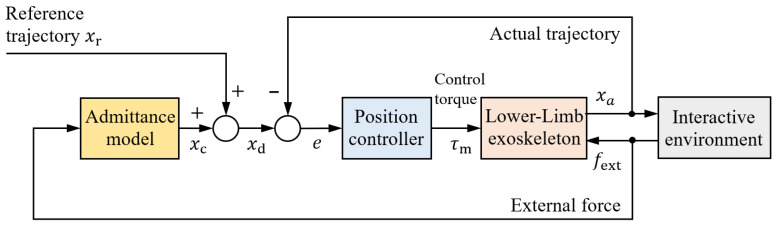
Admittance control structure.

Compliant control of exoskeleton joints is essential for achieving safe and comfortable human–robot interaction [84]. Unlike position control, which rigidly enforces the robot to follow predefined trajectories, compliant control dynamically regulates the relationship between human motion and external forces [85]. This is particularly important for accommodating individual variability and ensuring natural assistance during rehabilitation. Various compliant control algorithms have been developed [86,87], including impedance control (Figure 5), admittance control (Figure 6), and their adaptive or hybrid extensions, which adjust control parameters to maintain stable and compliant interaction. More recent advancements incorporate user intent estimation and skill-learning techniques to further improve interactive flexibility [88,89]. This section reviews the latest developments in these compliant control strategies.

### 5.1. Impedance Control

**Figure 7:**
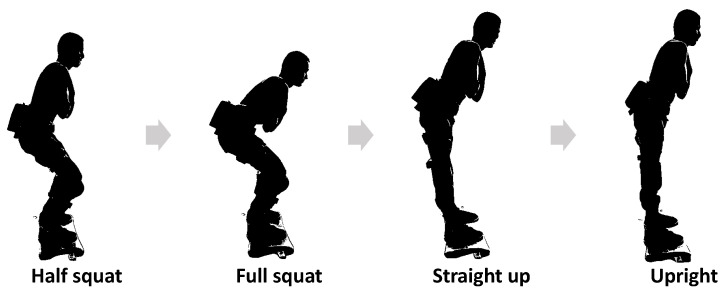
A human is performing a squat-like motions while wearing the exoskeleton.

**Figure 8:**
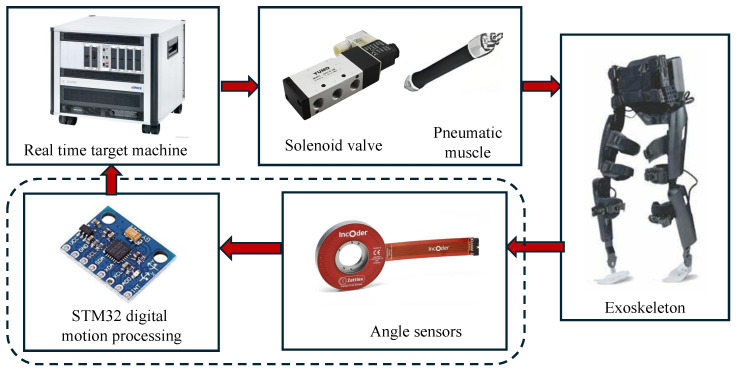
The hardware of the control architecture of the lower-limb exoskeleton.

Impedance control regulates a dynamic relationship between human motion and interaction forces, behaving like a virtual spring-damper system [90]. Building on this concept, an active impedance control is proposed to improve the kinematic response of the limbs as increasing the user’s average speed of motion [91]. A human-centered active torque-based assist-as-needed impedance control is developed for lower-limb patient--exoskeleton coupling system, which considers the coupling effects such as elastic connections and human soft tissues [92]. As demonstrated in Figure 7, impedance modulation in lower-limb exoskeletons optimizes sit-to-stand movement assistance by adapting the human–exoskeleton system’s mechanical impedance, thereby reducing required wearer effort [93]. In gait assistance applications, the impedance control is proposed for adjusting the trajectory of a lower-limb exoskeleton during the swing phase of walking, as shown in Figure 8 [94]. To adapt to the walking scenarios, a switching control strategy is given by combining impedance control with sliding mode control for a lower-limb exoskeleton [95]. The design of a rehabilitation exoskeleton incorporates impedance control, demonstrating its effectiveness in providing adaptive assistance during rehabilitation exercises [96]. Advanced integrations such as fuzzy logic-enhanced sliding mode control leverage time delay estimation to strengthen impedance control robustness in upper extremity rehabilitation robots [97]. In summary, impedance control has been extensively applied in exoskeleton systems to enhance adaptive assistance, optimize motion support, and ensure safe human-robot interaction across various tasks and anatomical regions. These advancements establish a solid foundation for exploring more advanced control strategies.

### 5.2. Admittance Control

Unlike impedance control, admittance control places the position loop in the inner layer, generating motion commands in response to external forces to achieve compliant interaction [98], which enhances control stability in the presence of uncertainties in both internal model and external environment. To enhance lower-limb assistance, researchers have developed admittance shaping controllers that dynamically adjust the exoskeleton’s response characteristics according to user requirements [99]. Building upon this concept, a coordinated admittance control framework has been recently proposed, which enables synchronized multi-joint assistance through coupled impedance parameter regulation [100]. In addition, an admittance shaping-based assistive control is implemented for a series elastic actuator driven robotic hip exoskeleton that can assist individuals with hip muscle weakness to restore normative mobility [101]. Electromyography (EMG)-based admittance control is integrated into a hybrid rehabilitation robot to dynamically modulate assistance levels. This approach effectively promotes patient engagement during arm and hand exercises [102]. Similarly, the admittance control is used to adjust the exoskeleton’s response based on user input, providing tailored support to improve motion control and rehabilitation outcomes [103]. Notably, recent studies integrate physiological cost evaluation into admittance control, validating rehabilitation efficiency by quantifying users’ metabolic responses during gait training [104]. These studies demonstrate that admittance control offers a flexible and user-responsive framework, enabling personalized and efficient assistance in lower-limb exoskeletons, with proven benefits in improving motion coordination, rehabilitation effectiveness, and physiological adaptability.

### 5.3. Adaptive Impedance/Admittance Control

**Figure 9:**
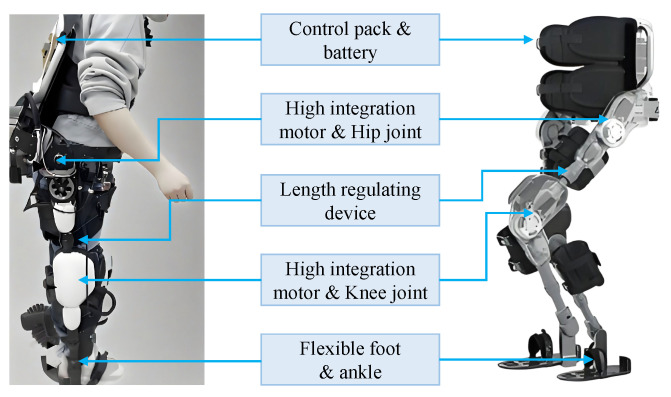
Testbed for adaptive impedance/admittance control with Fourier X2.

To cope with time-varying interactive environments, adaptive impedance/admittance control is increasingly used in exoskeleton assist joints. Adaptive sliding mode control is adopted to adjust admittance levels of lower-limb exoskeletons, optimizing assistance during rehabilitation exercises [105]. As shown in Figure 9, by optimizing impedance parameters using trajectory deviations, the authors of [106] ensured accurate tracking and minimal human–robot interaction forces, enhancing adaptability and assistance effectiveness under different tasks. The variable impedance control is used to modulate assistance provided by the exoskeleton during walking [107]. Through dynamically adjusting the impedance levels, the user adaptability and walking efficiency are enhanced. A cooperative control framework is introduced for a lower-limb exoskeleton using adaptive impedance control. By adjusting actuator stiffness and impedance gains based on human joint torque, the system achieves enhanced safety and tracking performance [108]. An adaptive backstepping sliding mode control strategy is introduced for a pediatric lower-limb exoskeleton. This subject-cooperative control method ensures precise and safe interaction [109]. To ensure that a smart and compliant system is developed, the target admittance gains of the controller is adapted according to the concept of energy. Such gains are modified so that an exoskeleton reduces interaction energy in cases wherein users have sufficient strength for task execution [110]. In summary, adaptive impedance and admittance control strategies dynamically adjust parameters based on trajectory deviations, joint torque, or energy-based criteria, thereby improving exoskeleton assistance, user adaptability, and safety across diverse rehabilitation tasks and users.

Impedance, admittance, and adaptive control each offer distinct advantages in enhancing human–robot interaction and user comfort. Impedance control provides direct and responsive interaction but is sensitive to parameter tuning. Admittance control ensures smooth and stable motion, though it may introduce latency and reduce transparency in dynamic tasks. Adaptive control enables real-time personalization and improved robustness across users and conditions, but at the cost of increased complexity. In practice, hybrid approaches are often adopted to combine the strengths of multiple methods for improved comfort, stability, and adaptability.

### 5.4. Hybrid Impedance/Admittance Control

Hybrid impedance/admittance control provides a balanced approach that leverages the strengths of both control strategies, enhancing adaptability, safety, performance, and robustness in robotic systems [111]. A hybrid control mode combines assist-as-needed control with adaptive adjustments to facilitate rehabilitation training for upper-limb exoskeletons [112]. An adaptive position constrained assist-as-needed control strategy adjusts the level of assistance based on the user’s position and effort, promoting active participation, and improving rehabilitation effectiveness [113]. A region-based barrier Lyapunov function is designed to separate the task workspace of the exoskeleton into a human region and a robot region, enabling the human leg to follow the desired motion trajectory. This achieves human-in-loop control for hemiplegia gait rehabilitation [114]. A series admittance–impedance controller has also been introduced to improve the robustness and stability of force control in robotic systems, enhancing the robot’s ability to handle varying force interactions more effectively [115]. Hybrid compliant control strategy is proposed for lower-limb exoskeletons to adapt to time-varying step frequencies. The controller enables user-intent-driven motion while enhancing human–exoskeleton coordination and wearing comfort [116]. These hybrid control strategies demonstrate significant potential in optimizing both safety and adaptability during rehabilitation.

Together, adaptive and hybrid strategies offer improved transparency, stability, and personalization, significantly enhancing human–robot interaction. These approaches are especially beneficial in lower-limb exoskeletons, where control demands vary with terrain, walking speed, and user condition.

### 5.5. Intent Estimation-Based Compliant Control

Intent estimation-based control infers the user’s movement intention in real time using signals such as joint kinematics or interaction forces. By proactively adjusting control parameters or generating desired trajectories, this approach enables more intuitive and responsive human–robot interaction, especially in dynamic or non-repetitive tasks [87,117]. For instance, an adaptive extended Kalman filter can compensate for trajectory errors in knee exoskeletons, thereby improving the human–exoskeleton interaction by accurately correcting deviations in the exoskeleton’s movement to match the intended trajectory [118]. Empirical evidence highlights that significant differences in sensor data before and after intent changes validate that changes in user intent do affect onboard sensor measurements during exoskeleton-assisted walking, which helps improve adaptive control strategies [119]. Multi-modal sensing improves intent recognition by combining physiological and biomechanical signals, enhancing accuracy and responsiveness-crucial for real-time, safe interaction in lower-limb exoskeletons. Systematic methods for characterizing, estimating, and implementing human intent in exoskeleton-assisted walking enhance the alignment between user intentions and exoskeleton responses, ensuring better assistance [120]. Notably, a neuro-adaptive controller estimates user intent in real time without relying on predefined models, thereby enhancing adaptability and responsiveness in human-robot interactions [121]. Furthermore, a comprehensive framework addresses both adaptation to the human partner and motion constraints, enabling safe and efficient physical human–robot interaction [122]. This paragraph synthesizes advancements in intent-aware robotics, emphasizing how adaptive algorithms and systematic frameworks bridge human intent with robotic responses. Key innovations include real-time estimation, sensor-driven validation, and dual optimization of adaptability and safety, collectively advancing human–exoskeleton collaboration.

### 5.6. Skill Learning-Based Compliant Control

**Figure 10:**
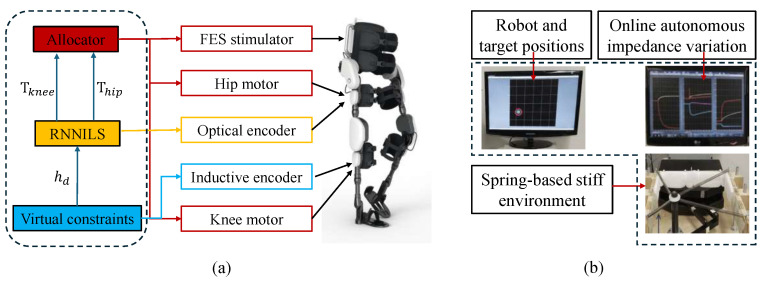
Testbeds for skill learning-based compliant control. (**a**) Exoskeleton testbed; (**b**) rehabilitation robot attached to a spring device.

**Figure 11:**
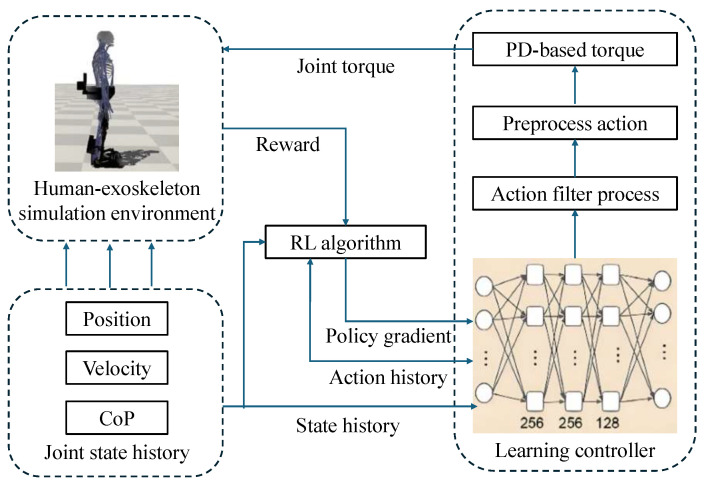
The overall learning process of the integrated robust motion controller.

Skill learning-based control utilizes data-driven methods to learn motion patterns or assistive strategies from demonstrations or interaction data. This allows exoskeletons to deliver personalized, adaptive assistance across users and contexts, moving beyond fixed-parameter control toward task-aware and user-specific behavior [123,124]. The cascaded impedance control is enhanced with iterative learning and integrates a fuzzy logic system using EMG and torque to adapt impedance in real time [125], improving safety, tracking, and responsiveness in rehabilitation exoskeletons. As shown in Figure 10a, a neural network-based iterative learning controller is proposed for the exoskeleton robot, enabling adaptive torque allocation between the robot and motors. This ensures accurate trajectory tracking, offering enhanced performance [126]. A control method incorporating model-free deep reinforcement learning, enables the exoskeleton to learn and adapt to optimal gait patterns autonomously, enhancing personalization and effectiveness [127]. A comprehensive framework integrating skill formalism, meta-learning, and adaptive control enables robots to efficiently learn and adapt to a wide range of manipulation tasks [128]. An adaptive skill learning method for contact-rich manipulation tasks, incorporates human feedback to refine and improve the robot’s compliant behaviors in real time [129]. As displayed in Figure 10b, adaptive control strategies based on impedance learning allows the robot to adjust its impedance dynamically in response to varying human actions, improving interaction safety and effectiveness [130]. A reinforcement learning-based controller is proposed for a lower-limb exoskeleton as shown in Figure 11. By incorporating center of pressure data into the control policy and reward, balance is effectively maintained [131]. This paragraph outlines recent advances in skill learning-based compliant control, highlighting the integration of machine learning techniques to enhance robotic adaptability and safety. These methods collectively advance the personalization and robustness of exoskeleton systems.

Machine learning enables real-time, data-driven adjustment of impedance or admittance parameters to match individual user needs. Supervised, reinforcement, and online learning approaches can personalize control strategies based on user features, interaction feedback, and changing conditions. This improves assistance quality, comfort, and adaptability in human-exoskeleton interaction.

## 6. Conclusions and Future Work

This survey discusses design and control methodologies of high-torque-density joints for compliant lower-limb exoskeleton. It firstly summarizes the design requirement of exoskeleton robots for lower-limb assistance. The exoskeleton system should generate high torque to support knee and hip movement with lightweight for comfortable wearing. Moreover, it is expected to have high bandwidth motion and back-drivability for flexible response to human body and external environment. Robustness is also important to improving the robot practicality user and experience in various situations.

To meet those requirements, the development of distinct types of actuator joints is discussed. The conventional joints with harmonic drive have high-output torque but high mechanical impedance, while recent developed series elastic joints have high flexibility but complex structure. In comparison, the quasi-direct-drive joints inherit the advantage of fast dynamic performance. Moreover, as the core component to drive joints, the permanent magnet synchronous motor (PMSM), especially axial flux permanent magnet machine (AFPMM), are widely utilized due to their compact structure and outstanding performance in output torque and efficiency. The recent advance regarding motor torque density, stability, efficiency, and safety is comprehensively discussed. Furthermore, different kinds of currently popular complaint control methods, including impedance control, admittance control, hybrid impedance/admittance control, adaptive impedance/admittance control, and intent estimation-based and skill learning-based compliant control, are analyzed to improve the robot ability in anthropomorphic movement and quick response.

The current design of joints is predominantly employed in lower-limb exoskeletons for flat ground walking. However, future designs should consider adaptability under complex terrain and working conditions, including energy regeneration and storage, and the release of external mechanical energy [132]. Thus, there are still numerous challenges expected to be overcome through future research. The introduction of series elastic actuation results in an increase in joint mass and structural complexity due to the addition of physical springs, as well as a reduction in output torque and limited bandwidth for control force and torque [133]. Quasi-direct joints exhibit higher torque density but are accompanied by higher Joule heat losses due to a larger current draw, motor mass and inertia issues, and specialized motor drivers are required for real-time commutation and control. Moreover, the cost of quasi-direct joints is high [56].

Further study will be conducted to research the influence of topological structure on the performance optimization for PMSM used in skeleton joints. According to the research reviewed in this survey, there are many structures with various parameters for PMSM which provide both challenges and prospects of drive motor development [134]. Not only the magnetic flux direction and stator/rotor structure but also the pole-slot ratio and air-gap length deeply impact the torque density, stability, efficiency, and safety of the motors [135]. To solve the optimization challenges for machines with multi-parameters and multi-optimization objectives, it is significant to establish the corresponding mathematic model which ranks the motor parameters according to their sensitivity of effect on certain optimization target [136]. Additionally, as the advancement of new iron core material and improvement in manufacture techniques, the electromagnetic and thermal characteristics of PMSM can be further researched [137]. Consequently, the improved motor is expected to provide skeleton joints with advanced capability in power, stability, and precision.

As for the development of complaint control, the primary challenge is achieving a precise and intuitive human–robot interaction [138]. It is exacerbated by the variability in human biomechanics and the limitations of current sensors, which can be prone to noise and drift, compromising performance [139]. To overcome such problems, future developments will likely focus on enhancing human–exoskeleton interaction through advanced sensing technologies and machine learning algorithms, such as integrating electromyography and inertial measurement units for real-time, detailed insights into user intented movement [140]. Developing flexible and adaptive control strategies, including hybrid systems combining impedance, admittance, and model predictive control, will be crucial for meeting diverse user needs [141,142]. Bio-inspired control methods that mimic natural human motor strategies can also improve effectiveness and comfort [143]. Efforts to miniaturize components and reduce cost through advanced materials and new topological design will be essential for making exoskeletons more affordable and accessible.

The current challenges and future research directions of mechanism design, motor optimization, and control strategies are summarized in Table 6, from which it can be concluded that the key problem in developing high-torque-density joints lies in balancing electromechanical performance, thermal safety, and integration constraints. Thus, the manufacture of modularized joints with a more compact structure and powerful output is required. While higher torque output is essential, it inevitably increases copper and iron losses, resulting in significant heat generation that may affect motor lifespan and user comfort. Although thermal management strategies such as phase-change materials and lumped thermal modeling have shown promise, they often increase system complexity and weight. Achieving compact, low-backlash, and high-efficiency transmission mechanisms remains a design bottleneck, especially when aiming to preserve dynamic responsiveness and back-drivability. Additionally, integrating such actuators with compliant control strategies requires stability under diverse gait patterns and variable loading conditions. To support real-world deployment, future modular joint designs must balance ease of manufacturing, cost-effectiveness, and maintainability without sacrificing performance, forming the foundation for scalable and robust exoskeleton solutions.

## Figures and Tables

**Table 1:** Movement pattern, range, and peak torque of human lower-limb joints.

Joint	Movement Pattern	Allowable Range	Walking Range	Peak Torque (Nm/kg)
hip	Stretching/flexion	$-125^{\circ }$∼$65^{\circ }$	$-25^{\circ }$∼$30^{\circ }$	1.2
knee	Stretching/flexion	$-130^{\circ }$∼$0^{\circ }$	$-65^{\circ }$∼$0^{\circ }$	0.5
ankle	Stretching/flexion	$-20^{\circ }$∼$45^{\circ }$	$-10^{\circ }$∼$20^{\circ }$	1.7

**Table 2:** Comparison of representative harmonic drive joints.

Exoskeleton	Actuator Type	Gear Ratio	Torque Output (Nm)	Total Mass (kg)
HAL Series	DC Motor + Harmonic Reducer	>50:1	−	∼15
GEMS	BLDC + Harmonic Reducer	75:1	14	2.9
USTC Rehabilitation	BLDC + Harmonic Reducer	160:1	62	−
UEXO Series Of UEST	DC Motor + Harmonic Reducer	101:1	29.7	2.4
Knee Assistant Exoskeleton	DC Motor + Harmonic Reducer	100:1	−	3.5

**Table 3:** Comparison of representative elastic joints.

Elastic Actuator Type	Key Components	Target Joint	Elastic Unit Design	Weight (kg)
Linear Series Elastic Actuator	Brushless DC motor, ball screw, encoder, compression spring	Knee (RoboKnee)	Compression spring	2.9
Linear Series Elastic Actuator	Custom linear actuator, torsional tandem spring	Knee (Mindwalke)	Torsional tandem spring	−
High-Power Series Elastic Joint	Frameless brushless motor, harmonic reducer, torque spring	Torque-controlled exoskeleton	Dual-spoke torque spring	−
Clutched Series Elastic Joint	Planar brushless motor, harmonic gearbox, electromagnetic clutch, disc torsion spring	Hip	Disc-shaped torsion spring	1.5
Parallel Elastic Actuator	Split structure, brushless DC motor, harmonic reducer, dual parallel springs	Universal	Dual parallel springs	2.34
Coupled Variable Parallel Elastic Actuator	Brushless motor, planar coil springs (parallel)	Hip (sagittal plane)	Planar coil springs	2.4
Hybrid Modular Actuator	Series-parallel elastic units	Knee	Composite elastic units	1.5
Hybrid Elastic Joint	Series module (brushless DC motor, planetary gear, butterfly spring) + parallel module (leaf spring)	Hip	Butterfly spring (series), leaf spring (parallel)	−

**Table 4:** Comparison of representative quasi-direct drive joints.

Design Case	Key Components	Target Joint	Technical Advantages	Limitations	Weight (kg)
Quasi-Direct Drive Joint	High-torque motor, embedded planetary gear, magnetic encoder, driver, controller	Multi-joint exoskeleton	Lightweight, high torque output, integrated control	Gear meshing backlash, heat dissipation	<1.0
Integrated Drive Module	Frameless motor, knee/ankle mechanical structure	Knee and ankle exoskeleton	Compact integration, power-intensive transmission	Limited torque scalability	−
Custom Motor Design	Frameless Custom BLDC motor with package windings, Sun-planet gear reducer	General joint applications	Enhanced torque density, shared support components	Complex manufacturing requirements	−
Rolling Knee Joint	Two-stage planetary gear reducer, synchronous belt, customized motor	Knee	Reduced inertia, distributed motor placement	Reduced motion accuracy	1.15

**Table 5:** Comparative analysis of joint design approaches for exoskeletons.

Joint Type	Torque Density	Back-Drivability	Dynamic Bandwidth	Mechanical Complexity
Harmonic Drive	Medium	Poor	Low	High
Series Elastic Actuator (SEA)	Low	Good	Moderate	High
Quasi-Direct Drive	High	Moderate	High	Moderate

**Table 6:** Summary of challenges and future research directions.

Aspect	Current Challenges	Future Research Directions
Mechanism design	Trade-off between joint compactness, alignment with human anatomy, and structural rigidity; limited modularity and adjustability.	Design of bio-inspired, lightweight, and modular joint mechanisms with better joint alignment and structural compliance.
Motor design	Difficulty achieving high torque density, efficiency, and thermal stability in compact spaces; limited customization for wearable use.	Development of high-torque-density motors optimized for wearable robotics; improved cooling and winding strategies.
Compliant control	Sensitivity to parameter tuning (impedance/admittance); limited robustness in real-world scenarios.	Learning-based adaptive control tailored to user states and terrain conditions; safe gain auto-tuning.

## Abbreviations

The following abbreviations are used in this manuscript:

ACSM	Alternative Current Servo Motor
AFIR	Axial Flux Internal Rotor
AFPMM	Axial Flux Permanent Magnet Machine
BLDC	Brushless Direct Current
DC	Direct Current
EMG	Electromyography
PMSM	Permanent Magnetic Synchronous Motor
LPTN	Lumped Parameter Thermal Network
L-SEA	Linear Series Elastic Actuation
PCM	Phase-Change Material
PEA	Parallel Elastic Actuator
PWM	Pulse Width Modulation
RFPMM	Radial Flux Permanent Magnet Machine
R-SEA	Rotary Series Elastic Actuation
UEST	University of Electronics Science and Technology
USTC	University of Science and Technology China
